# Identification of flowering genes in strawberry, a perennial SD plant

**DOI:** 10.1186/1471-2229-9-122

**Published:** 2009-09-28

**Authors:** Katriina Mouhu, Timo Hytönen, Kevin Folta, Marja Rantanen, Lars Paulin, Petri Auvinen, Paula Elomaa

**Affiliations:** 1 Department of Applied Biology, PO Box 27, FIN-00014 University of Helsinki, Helsinki, Finland; 2 Finnish Graduate School in Plant Biology, PO Box 56, FIN-00014 University of Helsinki, Helsinki, Finland; 3 Viikki Graduate School in Biosciences, PO Box 56, FIN-00014 University of Helsinki, Helsinki, Finland; 4 Horticultural Sciences Department, University of Florida, Gainesville, FL, USA; 5 Institute of Biotechnology, PO Box 56, FIN-00014 University of Helsinki, Helsinki, Finland

## Abstract

**Background:**

We are studying the regulation of flowering in perennial plants by using diploid wild strawberry (*Fragaria vesca *L.) as a model. Wild strawberry is a facultative short-day plant with an obligatory short-day requirement at temperatures above 15°C. At lower temperatures, however, flowering induction occurs irrespective of photoperiod. In addition to short-day genotypes, everbearing forms of wild strawberry are known. In 'Baron Solemacher' recessive alleles of an unknown repressor, *SEASONAL FLOWERING LOCUS *(*SFL*), are responsible for continuous flowering habit. Although flower induction has a central effect on the cropping potential, the molecular control of flowering in strawberries has not been studied and the genetic flowering pathways are still poorly understood. The comparison of everbearing and short-day genotypes of wild strawberry could facilitate our understanding of fundamental molecular mechanisms regulating perennial growth cycle in plants.

**Results:**

We have searched homologs for 118 *Arabidopsis *flowering time genes from *Fragaria *by EST sequencing and bioinformatics analysis and identified 66 gene homologs that by sequence similarity, putatively correspond to genes of all known genetic flowering pathways. The expression analysis of 25 selected genes representing various flowering pathways did not reveal large differences between the everbearing and the short-day genotypes. However, putative floral identity and floral integrator genes *AP1 *and *LFY *were co-regulated during early floral development. *AP1 *mRNA was specifically accumulating in the shoot apices of the everbearing genotype, indicating its usability as a marker for floral initiation. Moreover, we showed that flowering induction in everbearing 'Baron Solemacher' and 'Hawaii-4' was inhibited by short-day and low temperature, in contrast to short-day genotypes.

**Conclusion:**

We have shown that many central genetic components of the flowering pathways in *Arabidopsis *can be identified from strawberry. However, novel regulatory mechanisms exist, like *SFL *that functions as a switch between short-day/low temperature and long-day/high temperature flowering responses between the short-day genotype and the everbearing 'Baron Solemacher'. The identification of putative flowering gene homologs and *AP1 *as potential marker gene for floral initiation will strongly facilitate the exploration of strawberry flowering pathways.

## Background

Transition from vegetative to reproductive growth is one of the most important developmental switches in plant's life cycle. In annual plants, like *Arabidopsis*, flowering and consequent seed production is essential for the survival of the population until the following season. To assure timely flowering in various environments, *Arabidopsis *utilizes several genetic pathways that are activated by various external or internal cues. Light and temperature, acting through photoperiod, light quality, vernalization and ambient temperature pathways, are the most important environmental factors regulating flowering time [1]. Moreover, gibberellin (GA) and autonomous pathways promote flowering by responding to internal cues [2,3]. In contrast to annual plants, the growth of perennials continues after generative reproduction, and the same developmental program is repeated from year to year. Regulation of generative development in these species is even more complex, because other processes like juvenility, winter dormancy and chilling are tightly linked to the control of flowering time.

In *Arabidopsis *photoperiodic flowering pathway, phytochrome (phy) and cryptochrome (cry) photoreceptors perceive surrounding light signals and reset the circadian clock feedback loop, including TOC1 (TIMING OF CAB EXPRESSION), CCA1 (CIRCADIAN CLOCK ASSOCIATED 1) and LHY (LATE ELONGATED HYPOCOTYL) [4-7]. The central feature in the photoperiodic flowering is the clock generated evening peak of *CO *(*CONSTANS*) gene expression [8]. In long-day (LD) conditions, *CO *peak coincidences with light resulting in accumulation of CO protein in the leaf phloem and consequent activation of the expression of *FT *(*FLOWERING LOCUS T*) [9]. FT protein, in turn, moves to the shoot apex, and together with FD triggers floral initiation by activating floral identity gene *AP1 *(*APETALA 1*) [10,11]. *FT*, together with *SOC1 *(*SUPPRESSOR OF OVEREXPRESSION OF CONSTANS 1*) and *LFY *(*LEAFY*) form also convergence points for different flowering pathways, and therefore are called flowering integrator genes [12].

In winter-annual ecotypes of *Arabidopsis*, MADS-box gene *FLC *(*Flowering Locus C*) prevents flowering by repressing *FT *and *SOC1*, and vernalization is needed to nullify its function [13]. The major activator of *FLC *is FRI (FRIGIDA) [14], but several other proteins, including for example FRL1 (FRIGIDA-LIKE 1) [15], PIE (PHOTOPERIOD INDEPENDENT EARLY FLOWERING 1) [16], ELF7 and ELF8 (EARLY FLOWERING 7 and 8) [17], and VIP3 (VERNALIZATION INDEPENDENCE 3) [18] are also needed to maintain high *FLC *expression. During vernalization, *FLC *is down-regulated by VRN2-PRC2 (Vernalization 2 - Polycomb Repressive Complex 2) protein complex containing low temperature activated VIN3 (VERNALIZATION INSENSITIVE3), allowing plants to flower [19,20].

Autonomous and GA pathways respond to endogenous cues to regulate flowering time. The role of the autonomous pathway is to promote flowering by lowering the basal level of *FLC *transcription [3]. Autonomous pathway consists of few sub-pathways, which include for example RNA processing factors encoded by *FCA, FPA, FLK *(*FLOWERING LOCUS K*), *FY *and *LD *(*LUMINIDEPENDENS*) [21], putative histone demethylases *LDL1 *and *LDL2 *(*LSD1-LIKE 1 *and *2*) [22], and deacetylases *FLD *(*Flowering locus D*) and *FVE *[23,24]. GA pathway is needed to induce *LFY *transcription and flowering in short-day (SD) conditions [25].

Strawberries (*Fragaria *sp.) are perennial rosette plants, belonging to the economically important Rosaceae family. Most genotypes of garden strawberry (*Fragaria *× *ananassa *Duch.) and wild strawberry (*F. vesca *L.) are Junebearing SD plants, which are induced to flowering in decreasing photoperiod in autumn [26,27]. In some genotypes, flowering induction is also promoted by decreasing temperatures that may override the effect of the photoperiod [27,28]. In contrast to promotion of flowering by decreasing photoperiod and temperature, these "autumn signals" have opposite effect on vegetative growth. Petiole elongation decreases after a few days, and later, around the floral transition, runner initiation ceases and branch crowns are formed from the axillary buds of the crown [29,30]. Crown branching has a strong effect on cropping potential as it provides meristems that are able to initiate inflorescences [31].

In addition to SD plants, everbearing (EB) genotypes are found in garden strawberry and in wild strawberry [29,32]. Environmental regulation of induction of flowering in EB genotypes has been a topic of debate for a long time. Several authors have reported that these genotypes are day-neutral [29,33]. Recent findings, however, show that long-day (LD) accelerates flowering in several EB *Fragaria *genotypes [34,35]. Interestingly, in wild strawberry genotype 'Baron Solemacher' recessive alleles of *SFL *gene locus (*SEASONAL FLOWERING LOCUS*) have been shown to cause EB flowering habit [36]. *SFL *has not been cloned, but it seems to encode a central repressor of flowering in wild strawberry. Consistent with the repressor theory, LD grown strawberries have been shown to produce a mobile floral inhibitor that is able to move from mother plant to the attached runner plant [37]. GA is one candidate corresponding to this inhibitor, since exogenously applied GA has been shown to repress flowering in strawberries [38,39].

Identification of central genes regulating flowering time and EB flowering habit, as well as those controlling other processes affecting flowering, is an important goal that would greatly accelerate breeding of strawberry and other soft fruit and fruit species of Rosaceae family. In this paper, we have searched *Fragaria *homologs with the known *Arabidopsis *flowering time genes by EST sequencing and bioinformatics analysis. Dozens of putative flowering genes corresponding to all known genetic pathways regulating flowering time were identified. The expression analysis of several candidate flowering time genes revealed only few differences between the SD and EB wild strawberries, including the presence or absence of *AP1 *mRNA in the apices of EB and SD genotypes, respectively. Our data provides groundwork for detailed studies of flowering time control in *Fragaria *using transcriptomics, functional genomics and QTL mapping.

## Results

### Environmental regulation of flowering in two EB genotypes of wild strawberry

We studied the effect of photoperiod and temperature on flowering time in two EB genotypes, 'Baron Solemacher', which contains recessive alleles in *SFL *locus [40,41], and 'Hawaii-4'. Flowering time was determined by counting the number of leaves in the main crown before formation of the terminal inflorescence. In SD genotypes of the wild strawberry, SD (<15 h) or, alternatively, low temperature (~10°C) is needed to induce flowering [27]. In EB genotypes 'Baron Solemacher' and 'Rugen', instead, LD and high temperature has been shown to accelerate generative development [35], but careful analysis of the environmental regulation of flowering induction has so far been lacking.

**Figure 1:**
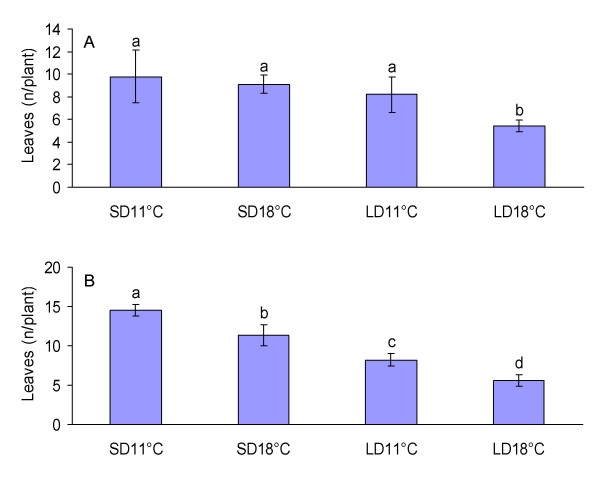
**Environmental regulation of flowering in everbearing wild strawberries**. The effect of photoperiod (SD 12 h, LD 18 h) and temperature (11/18°C) on the flowering time of 'Baron Solemacher' (A) and 'Hawaii-4' (B). Seeds were germinated in LD at 18°C, and seedlings were exposed to the treatments for five weeks, when the cotyledons were opened. After treatments, plants were moved to LD at 18°C and flowering time was recorded as number of leaves in the main crown before the terminal inflorescence. Values are mean ± SD. Pairwise comparisons between the treatments were done by Tukey's test, and statistically significant differences (*p *≤ 0.05) are denoted by different letters above the error bars.

Both 'Baron Solemacher' and 'Hawaii-4' produced five to six leaves in LD at 18°C before the emergence of the terminal inflorescence showing that they are very early-flowering in favorable conditions (Figure 1A and 1B). In 'Baron Solemacher', low temperature (11°C) or SD treatment for five weeks at 18°C clearly delayed flowering, but low temperature did not have an additional effect on flowering time in SD. Also in 'Hawaii-4', SD and low temperature delayed flowering, but all treatments differed from each other. Compared to the corresponding LD treatment, SD at 18°C doubled the number of leaves, and low temperature (11°C) delayed flowering time by about three leaves in both photoperiods. Thus, flowering induction in these EB genotypes is oppositely regulated by photoperiod and temperature than previously shown for the SD genotypes [27].

### Construction and sequencing of subtracted cDNA libraries

We constructed two subtracted cDNA libraries from LD grown EB genotype 'Baron Solemacher' and SD genotype, in order to identify differentially expressed flowering time genes in these genotypes. Plants were grown in LD conditions, where the SD genotype stays vegetative and the EB plants show early flowering. Pooled shoot apex sample covering the floral initiation period was collected from the EB genotype, and vegetative apices of the same age were sampled from the SD genotype. Suppression subtractive hybridization (SSH), the method developed for extraction of differentially expressed genes between two samples [42], was used to enrich either flowering promoting or flowering inhibiting transcripts from EB and SD genotypes, respectively.

A total of 1172 ESTs was sequenced from the library enriched with the genes of the SD genotype (SD library subtracted with EB cDNA) and 1344 ESTs from the library enriched with the EB genes (EB library subtracted with cDNA of the SD genotype). 970 SD ESTs [Genbank:GH202443-GH203412] and 1184 EB ESTs [GenBank:GH201259-GH202442] passed quality checking. Pairwise comparison of these EST datasets revealed that there was very little overlap between the libraries. However, general distribution of the sequences to functional categories (FunCat classification) did not reveal any major differences between the two libraries (Additional file 1).

BLASTx searches against *Arabidopsis*, Swissprot and non-redundant databases showed that over 70% of the ESTs gave a match in one or all of the three databases (Table 1). Moreover, tBLASTx comparison with different genomes revealed highest number of hits with *Populus trichocarpa *(Table 1). We also performed tBLASTx searches against TIGR plant transcript assemblies of *Malus *× *domestica*, *Oryza sativa *and *Vitis vinifera *and found hits for 64-76% of ESTs in these assemblies. Finally, the comparison of our sequences with a current *Fragaria *unigene list at the Genome Database for Rosaceae (GDR) showed that 38.2% of our ESTs are novel *Fragaria *transcripts. Taken together, depending on the analysis, 15-22% of sequences from SD genotype and 22-27% of EB sequences encode novel proteins, or originate from untranslated regions of mRNA. Moreover, the high number of novel *Fragaria *sequences in our libraries indicates that SSH method efficiently enriched rare transcripts in the libraries.

**Table 1:** The comparison of *F. vesca *ESTs with different databases.

		**WT**		**EB**	
		
		**number**	**average length**	**number**	**average length**
		
A)	Raw	1172	946	1344	965
	Poor Quality	202	1037	160	1066
	Singletons/ESTs	970	452	1184	451
	
		**number**	**%**	**number**	**%**
		
B)	Arabidopsis	695	72	781	66
	Swissprot	483	50	570	48
	Non-redundant	749	77	852	72
	In all 3 datab.	752	78	862	73
	
C)	Malus	741	76	874	74
	Oryza	689	71	807	68
	Vitis	666	69	761	64
	Populus	829	85	928	78
	
D)	No protein hits	218	22	322	27
	No Fragaria hits	370	38	454	38

Average numbers, lengths and percentages of ESTs from EB and SD genotypes. A) numbers and average lengths of raw and poor quality ESTs, and singletons, B) numbers and percentages of BLASTx hits against protein databases, C) numbers and percentages of tBLASTx hits against TIGR plant transcript assemblies of *Malus x domestica*, *Oryza sativa *and *Vitis vinifera *and against *Populus *genome database, D) numbers and percentages of novel ESTs.

### Identification of flowering time genes

**Figure 2:**
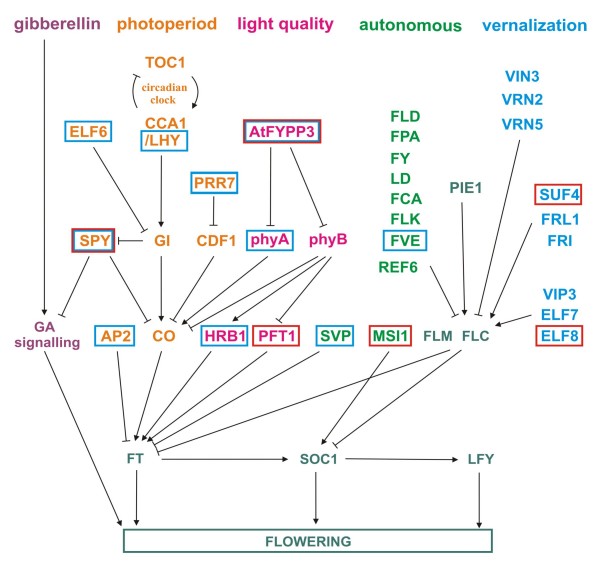
**A simplified chart showing *Arabidopsis *flowering pathways and corresponding gene homologs in *Fragaria***. Gene homologs found in cDNA libraries produced from SD and EB genotypes are surrounded by blue and red boxes, respectively. Arrows indicate positive regulation and bars negative regulation.

Flowering related genes were identified from our libraries by BLASTx searches as described above and fourteen putative flowering time regulators were identified; four gene homologs were present only in EB library, eight in SD library, and two genes in both libraries. In figure 2, we have summarized the *Arabidopsis *flowering pathways and highlighted the putative homologous genes identified from our EST collection. In general, candidate genes for all major pathways were identified. In addition, 118 *Arabidopsis *flowering time genes were used as a query to search publicly available GDR *Fragaria *EST and EST contig databases using tBLASTn. Sequences passing cut-off value of 1e-10 were further analysed by BLASTx algorithm against *Arabidopsis *protein database, and those returning original *Arabidopsis *protein were listed. Moreover, sequences that were absent from *Fragaria *databases were similarly searched from GDR Rosaceae EST database. In these searches, 52 additional *Fragaria *sequences were identified. Moreover, the total number of 88 homologs of *Arabidopsis *flowering time genes were found among all available Rosaceae sequences (Additional file 2).

Most genes of the *Arabidopsis *photoperiodic pathway were found also in *Fragaria*, and some of the lacking genes were present among Rosaceae ESTs (Table 2, Additional file 2). We found several genes encoding putative *Fragaria *photoreceptor apoproteins including phyA, phyC, cry2, ZTL (ZEITLUPE) and FKF1 (FLAVIN BINDING KELCH REPEAT F-BOX 1) [43]. Of the central circadian clock genes, homologs of *LHY *and *TOC1 *[5,7] were present in our EST libraries and GDR, respectively, but *CCA1 *[6] was lacking from both *Fragaria *and Rosaceae databases. Furthermore, a putative *Fragaria CO *from the flowering regulating output pathway has been cloned earlier [44]. Among the regulators of *CO *transcription and protein stability, GI (GIGANTEA) [45] was identified from Rosaceae and putative COP1, SPA3 and SPA4 [46,47] from *Fragaria*. In addition to genes of the photoperiodic pathway, homologs for both known sequences belonging to light quality pathways, *PFT1 *(*PHYTOCHROME AND FLOWERING TIME 1*) and *HRB1 *(*HYPERSENSITIVE TO RED AND BLUE 1*) [48,49], were found from our EST libraries.

**Table 2:** The list of genes belonging to the photoperiodic flowering pathway.

**Gene**	**AT gene locus**	**Biological function**	**Act./Repr. +/-**	**Reference**	**Fragaria**	**E-value**
*Photoreceptors and clock input*						

PhyA	AT1G09570	Red light photoreceptor	+	[78]	VES-002-C06	5E-33
PhyB	AT2G18790	Red light photoreceptor	-	[79]	nf	
CRY1	AT4G08920	Blue light photoreceptor	+	[79]	nf	
CRY2	AT1G04400	Blue light photoreceptor	+	[79]	DY669844	2E-110
ZTL	AT5G57360	F-box protein/blue light photoreceptor	+	[80]	EX668764	2E-97
FKF1	AT1G68050	F-box protein/blue light photoreceptor	+	[65]	DY671170	2E-54
ELF3	AT2G25920	Unknown	-	[60]	DY675323	3E-33
FYPP3	AT1G50370	Ser/Thr-specific protein phosphatase 2A	-	[81]	BAR-009-A02	1E-56
SRR1	AT5G59560	Unknown	-	[82]	CO817759	1E-10

*Circadian clock*						

LHY	AT1G01060	Myb domain TF	-	[7]	VES-005-E09	9E-19
CCA1	AT2G46830	Myb domain TF	-	[6]	nf	
TOC1	AT5G61380	Pseudo-response regulator	-	[5]	DY673134	1E-75
LUX	AT3G46640	Myb TF	-	[83]	DY668516	3E-43
ELF4	AT2G40080	Unknown	-	[84]	EX674323	2E-25
GI	AT1G22770	Unknown	+	[45]	nf	
PRR5	AT5G24470	Pseudo-response regulator	+	[85]	DY676242	3E-56
PRR7	AT5G02810	Pseudo-response regulator	+	[85]	VES-013-D12	5E-52
ELF6	AT5G04240	Jumonji/zinc finger-class TF	-	[86]	VES-002-F05	1E-45

*Output pathway*						

CO	AT5G15840	putative zinc finger TF	+	[8]	DY672035	2E-45
CDF1	AT5G62430		-	[65]	nf	
FT	AT1G65480	Phosphatidylethanolamine binding	+	[11]	nf	
TFL1	AT5G03840	Phosphatidylethanolamine binding	-	[87]	nf	
FD	AT4G35900	bZIP TF	+	[10]	EX675574	2E-14
COP1	AT2G32950	E3 ubiquitin ligase	-	[46]	DY667888	1E-94
SPA1	AT2G46340	WD domain protein	-	[47]	nf	
SPA3	AT3G15354	WD domain protein	-	[47]	DY671873	3E-24
SPA4	AT1G53090	WD domain protein	-	[47]	DY671245	2E-83
RFI2	AT2G47700	RIng domain zinc finger	-	[88]	nf	
HAP3b	AT5G47640	CCAAT-binding TF	+	[89]	EX658204	2E-60

The most important genes belonging to the photoperiodic pathway in *Arabidopsis *and their biological function are presented. Floral activators and repressors are indicated by + and - marks, respectively. Moreover, the presence or absence of homologous sequence in *Fragaria *sequence databases and E-value of BLASTx comparison against *Arabidopsis *are indicated. Sequences found in our libraries are named BAR and VES for everbearing genotype 'Baron Solemacher' and short-day genotype, respectively. Other ESTs and EST contigs are found from Genome Database for Rosaceae . More complete list is available in Additional file 2.

For the vernalization pathway, we were not able to find *FLC*-like sequences from our EST libraries or public *Fragaria *or Rosaceae EST databases by tBLASTn searches although we used the *FLC *and *FLC*-like sequences from *Arabidopsis *(*MAF1*-*MAF5, MADS AFFECTING FLOWERING 1-5*) and several other plant species as query sequences [13,50,51]. Similarly, also *FRI *[14] was lacking from Rosaceae ESTs but putative *FRL *(*FRIGIDA-LIKE*) [15] sequences were identified in *Fragaria*. In addition, we identified several gene homologs belonging to the FRI complex as well as other regulatory complexes (SWR1, PAF) involved in promoting the expression of *FLC *(Table 3, Additional file 2) [17,52,53]. Also putative members of *FLC *repressing PRC2 complex, were present in strawberry ESTs. These include putative *VIN3 *(*VERNALIZATION INSENSITIVE 3*) [19,20] that has been identified earlier [54], and putative *SWN1 *(*SWINGER 1*), *FIE *(*FERTILIZATION INDEPENDENT ENDOSPERM*), *VRN1 *(*VERNALIZATION 1*) and *LHP1 *(*LIKE HETEROCHROMATIN PROTEIN 1*) [19,55,56], which were found in this investigation (Table 3, Additional file 2). However, putative *VRN2 *that is needed for the repression of *FLC *by PRC2 was not found [19].

**Table 3:** The list of genes belonging to the vernalization pathway.

**Gene**	**AT gene locus**	**Biological function**	**Act./Repr. +/-**	**Reference**	**Fragaria**	**E-value**
FLC	AT5G10140	MADS-box TF	-	[13]	nf	
MAF1/FLM	AT1G77080	MADS-box TF	-	[50]	nf	

*Fri complex*						

FRI	AT4G00650	Unknown, enhancer of FLC	-	[14]	nf	
FRL1	AT5G16320	Unknown, enhancer of FLC	-	[15]	EX686406	4E-45
FRL2	AT1G31814	Unknown, enhancer of FLC	-	[15]	Contig 4768	6E-49
FES1	AT2G33835	CCCH zinc finger protein	-	[53]	nf	
SUF4	AT1G30970	putative zinc finger containing TF	-	[53]	BAR-003-F06	5E-46

*Swr complex*						

PIE	AT3G12810	ATP-dependent chromatin-remodelling factor	-	[16]	nf	
SEF1/SWC6	AT5G37055	Component of chromatin remodelling complex	-	[52]	DY670674	4E-70
ARP6/ESD1	AT3G33520	Component of chromatin remodelling complex	-	[52]	nf	
ATX1	AT2G31650	Putative SET domain protein	-	[90]	EX687477	4E-71

*Paf1 complex*						

ELF7	AT1G79730	RNA polymerase 2 associated factor 1 -like	-	[17]	nf	
ELF8	AT2G06210	RNA polymerase 2 associated factor -like	-	[17]	BAR-008-H08	3E-42
VIP4	AT5G61150	RNA polymerase 2 associated factor -like	-	[91]	EX660943	2E-50
VIP3	AT4G29830	RNA polymerase 2 associated factor -like	-	[18]	EX675781	7E-98
EFS/SDG8	AT1G77300	putative histone H3 methyltransferase	-	[53]	nf	

*VRN2-PRC2 complex*						

VRN2	AT4G16845	Polycomb group zinc finger	+	[92]	nf	
CLF	AT2G23380	Polycomb group protein	+	[93]	nf	
SWN1/EZA	AT4G02020	Polycomb group protein	+	[93]	EX687655	3E-114
FIE	AT3G20740	Polycomb group protein	+	[93]	DY671601	1E-112
VIN3	AT5G57380	PHD domain protein	+	[20]	CO816801	2E-58
LHP1	AT5G17690	epigenetic silencing	+	[56]	DY669633	2E-40
VRN1	AT3G18990	DNA binding protein	+	[55]	DY670727	8E-43

The most important genes belonging to the vernalization pathway in *Arabidopsis *and their biological function are presented. Floral activators and repressors are indicated by + and - marks, respectively. Moreover, the presence or absence of homologous sequence in *Fragaria *sequence databases and E-value of BLASTx comparison against *Arabidopsis *are indicated. Sequences found in our libraries are named BAR and VES for everbearing genotype 'Baron Solemacher' and short-day genotype, respectively. Other ESTs and EST contigs are found from Genome Database for Rosaceae . More complete list is available in Additional file 2.

In addition to the photoperiod and the vernalization pathways, we searched candidate genes for the autonomous and GA pathways. Several sequences corresponding to *Arabidopsis *genes from both pathways were identified suggesting the presence of these pathways also in *Fragaria *(Table 4, Additional file 2). Among these genes we found homologs for *Arabidopsis FVE *and *SVP *which have been shown to control flowering in a specific thermosensory pathway [24,57]. Moreover, some additional flowering time regulators that are not placed to any specific pathway were identified (Table 4, Additional file 2).

**Table 4:** The list of genes belonging to autonomous and gibberellin flowering pathways.

**Gene**	**AT gene locus**	**Biological function**	**Act./Repr. +/-**	**Reference**	**Fragaria**	**E-value**
*Autonomous pathway*						

FCA	AT4G16280	RRM-type RNA binding domain containing	+	[94]	nf	
FPA	AT2G43410	RRM-type RNA binding domain containing	+	[95]	nf	
FLK	AT3G04610	KH-type RNA binding domain containing	+	[96]	EX668302	5E-52
FY	AT5G13480	mRNA 3' end processing factor	+	[97]	EX659635	5E-75
SKB1	AT4G31120	Arginine methyltransferase	+	[98]	nf	
FVE	AT2G19520	retinoblastoma associated	+	[24]	VES-001-B03	3E-76
LD	AT4G02560	DNA/RNA binding homeodomain protein	+	[99]	DY670534	3E-49
FLD	AT3G10390	component of histone deacetylase complex	+	[23]	nf	
LDL1/SWP1	AT1G62830	Histone H3-Lys 4 demetylase-like	+	[22]	Contig 2573	2E-27
LDL2	AT3G13682	Histone H3-Lys 4 demetylase-like	+	[22]	DY669828	1E-42

*Gibberellin pathway*						

GAI	AT1G14920	putative transcriptional repressor	-	[100]	Contig 3276	3E-147
RGA	AT2G01570	putative transcriptional repressor	-	[100]	DQ195503	8E-60
SPY	AT3G11540	O-linked N-acetylglucosamine transferase	-	[101]	BAR-002-C02	2E-93
DDF1	AT1G12610	AP2-like TF	+	[102]	Contig 3158	5E-49
DDF2	AT1G63030	AP2-like TF	+	[102]	nf	
AtMYB33	AT5G06100	MYB TF	+	[25]	DY669997	5E-29
FPF1	AT5G24860	Unknown	+	[103]	Contig 4074	7E-38

*Other*						

SVP	AT2G22540	MADS-box TF	-	[57]	VES-013-D05	5E-22
AP2	AT4G36920	AP2 TF	-	[104]	VES-008-A07	9E-16
PFT1	AT1G25540	vWF-A domain protein	+	[48]	BAR-002-D08	1E-17
HRB1	AT5G49230	ZZ type zinc finger protein	+	[49]	VES-012-B01	7E-22

The most important genes of *Arabidopsis *autonomous and gibberellin pathways as well as some other floral regulators are presented. The biological function of the genes is indicated, and floral activators and repressors are marked by + and - marks, respectively. Moreover, the presence or absence of homologous sequence in *Fragaria *sequence databases and E-value of BLASTx comparison against *Arabidopsis *are indicated. Sequences found in our libraries are named BAR and VES for everbearing genotype 'Baron Solemacher' and short-day genotype, respectively. Other ESTs and EST contigs are found from Genome Database for Rosaceae . More complete list is available in Additional file 2.

### Identification of floral integrator genes in *Fragaria*

**Figure 3:**
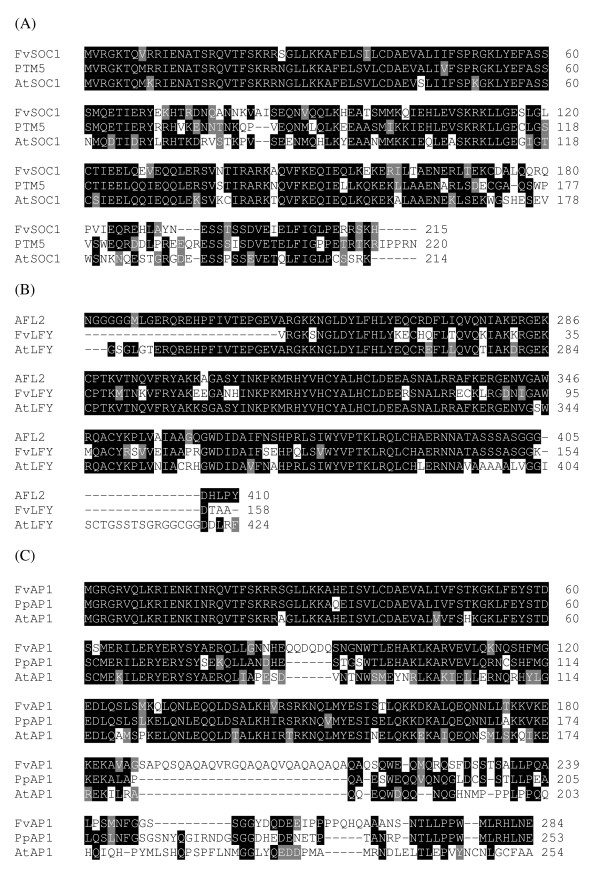
**Protein alignments of *Fragaria *flowering integrator and identity genes**. Multiple alignments of *Fragaria *protein sequences of full length SOC1 (A), partial LFY (B) and full-length AP1 (C) with closest protein homologs and corresponding protein sequence of *Arabidopsis thaliana*. Alignments were done by ClustalW (A, B) or T-Coffee (C) and modified by Boxshade program. *F. vesca *AP1 protein sequence was translated from GDR *Fragaria *EST contig 4941. PTM5 = *Populus tremuloides *MADS5, AFL2 = Apple FLORICAULA 2, PpAP1 = putative *Prunus persica *AP1.

Sequencing of our EST collections did not reveal any homologs for the floral integrator or identity genes such as *FT*, *SOC1*, *LFY *or *AP1 *[12,58]. A full-length cDNA sequence of *SOC1 *homolog [GenBank:FJ531999] and a 713 bp 3'-end fragment of putative *LFY *[GenBank:FJ532000] were isolated using PCR. Closest protein homolog of the putative FvSOC1 was 72% identical *Populus trichocarpa *MADS5, and the putative FvLFY showed highest amino acid identity (79%) to *Malus domestica FL2*. Comparison to *Arabidopsis *showed that AtSOC1 and AtLFY, respectively, were 66% and 75% identical with the corresponding wild strawberry protein sequences (Figure 3A and 3B). *FT *homolog, instead, was not identified in *Fragaria *despite of many attempts using degenerate PCR and screening of cDNA library plaques and *E.coli *clones from a variety of tissues and developmental conditions with the *Arabidopsis *coding sequence (K. Folta, unpublished). However, a putative *FT *was found in *Prunus *and *Malus *protein databases at NCBI. Among the other genes belonging to the same gene family, homologs of *MFT *(*MOTHER OF FT AND TFL1*) and *ATC *(*ARABIDOPSIS CENTRORADIALIS*) [59] were present in GDR *Fragaria *EST. Moreover, an EST contig corresponding to the floral identity gene *AP1 *was found. The length of the translated protein sequence of *FvAP1 *was 284 amino acids, being 30 amino acids longer than the corresponding *Arabidopsis *sequence. However, *FvAP1 *EST contig contained an unknown sequence stretch of 81 bp at nucleotide position 596-677. Putative FvAP1 showed highest overall identity (68%) with putative AP1 from *Prunus persica *(Figure 3C). Moreover, the 5' sequence containing 187 amino acids (the sequence before the unknown part) was 73% identical with the *Arabidopsis *AP1.

### Gene expression analysis revealed few differences between EB and SD genotypes

We compared the expression of selected flowering time genes (Table 5) corresponding to each flowering pathway in the leaf and shoot apex samples of EB and SD genotypes in order to explore the role of different pathways. Only few of the analysed genes were differentially expressed between the genotypes. Floral integrator gene *LFY *was slightly up-regulated in the shoot apex samples of EB (Table 6). Moreover, PCR expression analysis with two different primer pairs showed that *AP1 *was specifically expressed in EB apices correlating with the identity of the meristems. Among the genes from different flowering pathways, only two genes, vernalization pathway gene *ELF8 *[17] and photoperiod pathway gene *ELF3 *[60], were slightly differentially expressed between the genotypes (Table 6).

**Table 5:** The list of PCR primers used in real-time RT-PCR.

**Gene**	**Forward primer**	**Reverse primer**
*UBI*	CAGACCAGCAGAGGCTTATCTT	TTCTGGATATTGTAGTCTGCTAGGG
*LFY*	CGGCATTACGTTCACTGCTA	CCTGTAACACGCCTGCATC
*SOC1*	CAGGTGAGGCGGATAGAGAA	AGAGCTTTCCTCTGGGAGAGA
*AP1*	CGCTCCAGAAGAAGGATAAGG	CATGTGACTGAGCCTGTGCT
*AP1*	TCTGAAGCACGTAAGGTCTA	ATCCTGATCATAACCTCCAG
*LHY*	AAAGCTGGAGAAGGAGGCAGTC	CCGAGGATAAGGATTGCTTGGT
*ZTL*	TGCATGGGGTAGTGAAACAA	CACCTCCGACAGTGACCTTT
*FKF1*	ACCCACATCGTTTGTGGTCT	ACATCAGGATCCACCAGAGG
*ELF3*	TCCTCCAAGGAACAAGATGG	CCATTCCCCTGATTTGAGAG
*ELF6*	TTCGAAGGTCTTGGCAATGG	GCGCCTGAGTTTTATCCAACAC
*COL4*	GACCGAGAAATCCACTCTGC	CTCTCCGTCCGACAAGTAGC
*CO*	GACATCCACTCCGCCAAC	GTGGACCCCACCACTATCTG
*PFT1*	GCGACATGCCAAGGTTAGAATT	TCAGCGCCTCACACTCTTACAC
*HRB1*	GAATGGTGGACATCAGCAATCC	CCTCCGAAAGAATTGCTCAACA
*FYPP3*	ACAAAATGGCCCCTCATGTG	TGTGCTATGTGTCCATGGTGGT
*FRL*	CGCTAGTCAAGGTCGAGGAG	CGACTTCATCTCCATCAGCA
*ELF8*	GCTCAGAATGCTCCTCCTGT	TGAGTATTGCAGCCACTTGC
*VRN5*	AGCCCTTGATGTCATCAGCTG	CCGATGAATGGTTGGCTAATG
*MSI1*	TCTCCACACCTTTGATTGCCA	ACACCATCAGTCTCCTGCCAAG
*LHP1*	GGAGAGCCAGAACCAGGAG	CTCACCTTCTTCCCCTTCCT
*FVE*	GATCCAGCAGCAACCAAGTCTC	CCTCTTGGTGCAACAGAAGGAC
*SVP*	CGTGCTAAGGCAGATGAATGG	TGAAGCACACGGTCAAGACTTC
*SPY*	TGCGGTGTCAAATTGCATCA	GGCAACACTCAAGATGGATTGC
*GA3ox*	CCTCACAATCATCCACCAATCC	CGCCGATGTTGATCACCAA
*GA2ox*	CACCATGCCCAGAGCTTCA	AGGCCAGAGGTGTTGTTGGAT
*TFL1*	TGCAGAAACAAACGAGTTCGG	CCAAGAGCATCGATCATTTGGT
*AP2*	CCCGAAATCCTTGATTGTTCC	AACACTGCAATCGAACAACAGC

T_m_value of the primers is 60 ± 1°C.

**Table 6:** The expression of selected genes in the wild strawberry.

**Gene**	***MSI1 *as a control**	***FVE *as a control**
*Shoot apex samples*		
*AP1*	Expressed only in EB	Expressed only in EB
*LFY*	1.8 ± 0.4	1.9 ± 0.3
*ELF8*	1.5 ± 0.1	1.6 ± 0.1
*Leaf samples*		
*ELF3*	1.5 ± 0.1	1.8 ± 0.0

Relative gene expression in the shoot apex or leaf samples of LD grown plants of EB genotype compared to SD genotype. Ct values of genes of interest were normalized against Ctvalues of *MSI1 *and *FVE *to get normalized ΔCt values. The expression ratios between genotypes (EB/SD) were calculated from the formula 2^ΔCtEB^/2^ΔCtSD^. Values are mean ± standard deviation. Pooled shoot apex samples and leaf samples at four leaf stage were used.

### Developmental regulation of floral integrator, floral identity, and GA pathway genes

**Figure 4:**
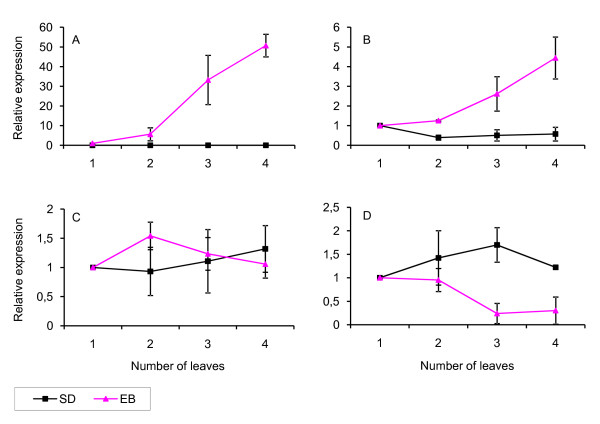
**Developmental regulation of gene expression in wild strawberry shoot apices**. The expression of *AP1 *(A), *LFY *(B), *SOC1 *(C) and *GA3ox *(D) in the SD and EB ('Baron Solemacher') genotype of the wild strawberry. Triplicate shoot apex samples were collected from LD grown plants at one to four leaf stage. Ct values were normalized against a *Ubiquitin *[GenBank:DY672326] gene to get normalized ΔCt values. The expression differences between one leaf stage and later developmental stages were calculated from the formula 2^ΔCt later developmental stage^/2^ΔCt one leaf stage^. The expression values at one leaf stage were artificially set to 1 separately for both genotypes. Values are mean ± SD. Note that *Ubiquitin *was amplified ~1 cycle earlier in SD genotype, but was stable between different developmental stages. Therefore, expression values between genotypes cannot be directly compared, while the expression levels between the various developmental stages are comparable.

We analysed the developmental regulation of *AP1*, *LFY*, *SOC1*, *GA3ox *and *GA2ox *transcription in the shoot apices of LD grown plants of EB and SD genotype containing one to four leaves. *Ubiquitin*, used as a control gene, was stable between different developmental stages, but was amplified ~1 PCR cycle earlier in SD genotype (Additional file 3). Thus direct comparison between the genotypes is not possible, but the trends during development are comparable. Three genes, *AP1*, *LFY*, and *GA3ox*, had clear developmental stage dependent expression pattern in EB apices, showing biggest changes after one or two leaf stage (Figure 4). The expression of *AP1 *was detected in EB apices already at one leaf stage, and its mRNA accumulated gradually reaching 6-fold increase at two leaf stage and 50-fold increase at four leaf stage (Figure 4A). In parallel, transcription of *LFY *started to increase at 2-leaf stage, but the change in its expression was much smaller (Figure 4B). A floral integrator gene, *SOC1*, in contrast, did not show clear developmental regulation (Figure 4C). Also GA pathway was co-regulated with *AP1 *and *LFY*, since GA biosynthetic gene *GA3ox *was strongly down-regulated after two leaf stage (Figure 4D). In addition, GA catabolism gene, *GA2ox*, tended to follow changes in the expression of *GA3ox*, although the results were not so clear (data not shown). In SD genotype, in contrast, *AP1 *was absent and other genes did not show clear developmental regulation (Figure 4). In this experiment, control plants of EB genotype flowered very early, after producing 4.7 ± 0.3 leaves to the main crown, whereas plants of SD genotype remained vegetative.

## Discussion

### Identification of flowering genes in strawberry

Genetic regulation of flowering in strawberry has earlier been studied only by crossing experiments. According to Weebadde et al. [61], everbearing character is a polygenic trait in garden strawberry whereas other studies indicate the presence of a single dominant gene [62]. Different results may arise from different origin of everbearing habit, since at least three different sources have been used in strawberry breeding [32,61,62]. Studies in *F. vesca *'Baron Solemacher'have shown that EB flowering habit in this genotype is controlled by recessive alleles of a single locus, called *seasonal flowering locus *(*sfl*) [40,41]. Identification of central genes regulating flowering, as well as those controlling other processes that affect flowering (runnering, chilling), is an important goal that would greatly accelerate breeding of strawberry and other soft fruit and fruit species of Rosaceae family.

For comprehensive identification of candidate genes of the strawberry flowering pathways, we searched homologs for 118 *Arabidopsis *flowering time genes from our own cDNA libraries and from GDR. In total, we were able to identify 66 gene homologs among about 53000 EST sequences. Moreover, gene homologs lacking from *Fragaria *were further mined from Rosaceae EST collections containing about 410 000 EST sequences. These searches revealed 22 additional putative flowering time genes in Rosaceae. Ongoing genome sequencing projects in apple, peach and wild strawberry will ultimately reveal the currently lacking flowering regulators in these species [63].

Sequences found in *Fragaria *corresponded to all known *Arabidopsis *flowering time pathways [2] suggesting that all of these genetic pathways may be present in *Fragaria*. However, the sequence conservation does not necessarily mean functional conservation, so major candidate genes from different pathways have to be functionally characterized in order to prove the presence of these pathways in strawberry. Few central regulators of flowering time are lacking from *Fragaria *sequence collections and some of them also from Rosaceae databases. For example, we were not able to identify a homolog for the florigen gene *FT *[11] in *Fragaria *regardless of several different attempts. This is probably due to its low expression level and tissue specific expression pattern [64]. Similarly, *GI*, which links circadian clock and *CO *[8,65], was absent from the *Fragaria *sequences. *FT *and *GI *homologs were, however, found in apple and *Prunus*, showing that they are present in Rosaceae. Moreover, consistent with studies in model legumes [66], *CCA1 *was lacking in Rosaceae, but its redundant paralog, *LHY*, is represented by few ESTs in *Fragaria*. CCA1 and LHY are MYB-type transcription factors which repress the expression of *TOC1 *in the central loop of *Arabidopsis *circadian clock [67]. Thus, in *Fragaria *and other species of Rosaceae family, LHY alone may control the expression of *TOC1 *in the clock core. This contrasts with other species, like *Populus*, where duplications of the *LHY/CCA1 *genes contribute to an apparently more complex mode of clock control [68].

Vernalization pathway in *Arabidopsis *culminates in *FLC *and *FLC*-like floral repressors [13,50]. They have been functionally characterized only in Brassicaceae [13,69], although homologous MADS box genes have been recently found from several eudicot lineages by phylogenetic analysis [51]. However, we were not able to identify *FLC*-like sequences in Rosaceae by using several *FLC*-like sequences as a query. Similarly, also *FRI *homologs were lacking from the Rosaceae sequence collections. However, putative homologs of *FRI*-like genes, *FRL1 *and *FRL2*, which are involved in *FLC *activation in *Arabidopsis *[15] were found, as well as several other homologs of genes belonging to *FLC *regulating protein complexes. Despite the presence of these transcripts, the presence of *FLC *is unclear, since at least PRC2 complex has several target genes [70]. Cloning and characterization of putative *FLC*-like and *FRI *genes as well as *FT *in strawberry would greatly expand our understanding of strawberry flowering pathways, and therefore, it is one of the most important targets of further studies. If these transcripts are present in strawberry, it is likely that the precise control of flowering has placed their expression in specific tissues or contexts where they are not easily detected. However, their presence should be substantiated in analysis of the impending genome sequence. Another important goal is the identification of putative Rosaceae or *Fragaria *specific flowering time genes. Ultimately, transcriptomics studies and functional analysis of central genes may reveal how different flowering pathways, which may be closely related to *Arabidopsis *pathways, make seasonal flowering in strawberry.

### What is the *SFL *gene?

*SFL *is a single dominant locus that enforces seasonal flowering habit in wild strawberry, and homozygous mutation in this locus leads to continuous flowering habit in at least one genotype, 'Baron Solemacher' [36]. In SD genotypes of wild strawberry, SD or low temperature induce flowering [27] probably by overcoming the function of *SFL *repressor gene. We showed here that EB genotypes 'Baron Solemacher' and 'Hawaii-4' produce only 5 - 6 leaves to the main crown before the formation of the terminal inflorescence in LD at 18°C. Hence, flowering induction in these conditions occurs soon after germination. In SD (12 h) or at low temperature (11°C) instead, plants formed several leaves more before the inflorescence. This finding shows that, in contrast to SD genotypes, both SD and low temperature restrain flowering induction in these genotypes, confirming earlier suggestions that EB genotypes of wild strawberry are in fact LD plants [35]. Most simple explanation for these opposite environmental responses is that the lack of flowering inhibitor, produced by active *SFL *gene, unmasks LD induced flowering promotion pathway in 'Baron Solemacher' and possibly in other EB genotypes. Given that both SD and low temperature repress *SFL*, analogous flowering regulating pathway has not yet been characterized at molecular level.

Our gene expression analysis did not give any hints of the putative location of *SFL *in wild strawberry flowering pathways. However, homologs of certain flowering repressors can be consireded as candidates for *SFL*, including the rice *CO *homolog *HD1 *(*HEADING DATE 1*), or *Arabidopsis *vernalization pathway genes *FLC *and *FRI *[13,14,71]. In strawberry, the role of vernalization pathway remains unclear until the presence or absence of FRI or FLC function is confirmed or other targets for this pathway are found. Strawberry *CO*, instead, has been cloned and mapped in *Fragaria *reference map, but its position does not match with the genomic location of *SFL *showing that *CO *itself is not *SFL *[44,72]. However, the possibility that some regulator of *CO *transcription or protein stability could be SFL cannot be ruled out and should be studied further.

Exogenously applied GA inhibits flowering in wild strawberry, and therefore, GA has been suggested to be a floral repressor [38,39]. Similar patterns have been observed and delineate differences between recurrent and non-recurrent roses [73]. However, we did not find clear differences in the expression of GA biosynthetic and catabolism genes, *GA3ox *and *GA2ox*, in the shoot apex samples of EB and SD genotypes before the floral initiation had occurred. In contrast, *GA3ox *was strongly repressed in EB apices after floral initiation and *GA2ox *showed similar trend. The fact that these changes in GA pathway occurred after two leaf stage suggests that GA signal was regulated during early flower development rather than during floral transition. These data does not support the role of endogenous GA as the regulator of flowering induction, indicating that *SFL *is not situated in the GA pathway. However, quantitative analysis of GA levels is needed to show whether the observed changes in the expression of GA pathway genes are reflected at the metabolic level.

### *AP1 *is a potential marker of floral initiation in strawberry

Gene expression analysis revealed that two putative flowering genes, *AP1 *and *LFY*, were co-regulated during floral development in EB wild strawberry. The homolog of floral identity gene *AP1 *was expressed in the EB apex already at one leaf stage, and its expression was strongly enhanced during later developmental stages. Also *LFY *mRNA accumulated along with *AP1 *during floral development in EB genotype, whereas *SOC1 *did not show a clear trend. The mRNA of *SOC1 *and *LFY *were present also in SD genotype, but *AP1 *transcription was not detected. In *Arabidopsis*, LFY and AP1 activate each other's expression constituting a feedback loop [12,58]. Moreover, *AP1 *is activated by FT-FD heterodimer shortly after flowering induction [10]. Thus, the expression patterns of *AP1*and *LFY *in the meristems of EB genotype suggest that flowering induction in these plants occurs before two leaf stage in LD conditions. Consistent with this conclusion, flower initials were clearly visible by stereomicroscope in the meristems at three or four leaf stage, and plants flowered after producing on average 4.7 leaves in the main crown. Based on our results, *AP1 *can be used as a marker for floral initiation in wild strawberry. However, functional studies are needed to confirm the role of *AP1*, *LFY *and *SOC1 *as floral integrator and identity genes, and this approach is currently going on.

## Conclusion

We have explored putative components for the genetic flowering pathways in perennial SD plant wild strawberry by identifying 66 homologs of *Arabidopsis *flowering time genes. Although few central genes are lacking, these data indicate that all known genetic flowering pathways may be present in *Fragaria*. This is consistent with the finding that EB genotypes, 'Hawaii-4' and 'Baron Solemacher', show similar environmental regulation of flowering than *Arabidopsis *summer-annuals. We also studied the expression of selected candidate genes and found that few genes were co-regulated in the shoot apex of the EB genotype during early floral development. Most strikingly, the mRNA of *AP1 *specifically accumulated in EB genotype, but was absent in SD genotype, showing its usefulness as a marker of floral initiation. Finally, identification of putative flowering time genes reported here enables their transcriptional and functional characterization, as well as genetic mapping, which may give answers for the relative importance of each genetic flowering pathway and lead to cloning of the central repressor gene, *SFL*. Ultimately, these genetic resources could be utilized in cultivar breeding of various species of Rosaceae family through genetic transformation and marker assisted selection breeding.

## Methods

### Plant materials, growing conditions and sampling

Seeds of SD and EB ('Baron Solemacher') genotypes of the wild strawberry (NCGR accession numbers [PI551792] and [PI551507], respectively) were sown on potting soil mixture (Kekkilä, Tuusula, Finland) and grown in a greenhouse under LD conditions (day length min. 18 h), provided by 400 W SON-T lamps (Airam, Kerava, Finland) and natural sunlight. After two to three leaves had developed per plant, shoot apex samples (tip of the shoot containing the meristem as well as two to three leaf initials) were collected under a stereomicroscope at ten different time points with three days intervals. Samples from each time point were pooled and used for the construction of cDNA libraries and real-time RT-PCR. WT samples contained shoot apices of the main crown, collected from 50 plants per time point. Also in EB genotype, shoot apices of the main crown were collected until the sepal initials became visible in the meristems. After this time point, the apices from one to three side shoots per plant were collected, altogether from 40 plants per sampling. In addition, leaf samples were collected from the same plants at four leaf stage for real-time RT-PCR analysis. Moreover, separate shoot apex samples were collected from WT and EB genotypes at one, two, three and four leaf stages. Control plants were grown in LD and their flowering time was determined by counting the number of leaves in the main crown before the terminal inflorescence. All samples were collected in July - August 2006 - 2008.

### Preparation and sequencing of subtracted cDNA libraries

Total RNA from pooled shoot apex samples was extracted with a pine tree method for RNA isolation [74]. The cDNA was synthesized with BD SMART cDNA Synthesis kit (Clontech, Palo Alto, US), amplified with PCR as instructed for subtraction, purified with Chroma Spin-1000 DEPC-H2O Columns (Clontech), extracted with chloroform:isoamylalcohol (24:1) using Phase Loch Gel Heavy 1.5 ml tubes (Eppendorf, Hamburg, Germany), digested with RsaI (Boehringer Mannheim, Mannheim, Germany), and purified with High Pure PCR Product Purification kit (Roche Diagnostics, Indianapolis, US). The cDNAs were subtracted using BD PCR-Select cDNA Subtraction Kit (Clontech) in both forward and reverse directions. The forward and reverse PCR mixtures were digested with RsaI (Boehringer Mannheim) and purified with High Pure PCR Product Purification Kit (Roche). After digestion, A-tailing was done as instructed in the technical manual of pGEM-T and pGEM-T Easy Vector Systems and PCR mixtures were ligated to pGEM-T Easy Vector (Promega, Wisconsis, US), and electroporated to TOP10 cells. The libraries were sequenced at the Institute of Biotechnology, University of Helsinki, as described earlier [75].

### Bioinformatics analysis

Raw EST sequences were quality checked before annotation. Base calling, end clipping and vector removal were performed by CodonCodeAligner-software (CodonCode Corporation, US). After this the ESTs were manually checked and sequences that contained poly-T in the beginning followed by short repetitive sequences were removed. BLASTx was performed against functionally annotated *Arabidopsis *protein database (v211200, MIPS), Swissprot and non-redundant protein database (NCBI), and *Populus trichocarpa *genome of DOE Joint Genome Institute [76] using cut-off value 1e-10. tBLASTx was performed against TIGR plant transcript assemblies of *Malus x domestica*, *Oryza sativa *and *Vitis vinifera *[77], and GDR *Fragaria *and Rosaceae Contigs using cut-off value 1e-10. For MIPS BLAST hits corresponding functional classes and Gene Ontology classes were obtained from Functional Classification Catalogue (Version 2.1) and GO annotation for *Arabidopsis thaliana *(Version 1.1213).

Homologs of Arabidopsis flowering time genes were searched from GDR *Fragaria *contig and EST databases using tBLASTx algorithm and *Arabidopsis *protein sequences as a query. Homologous sequences passing a cut-off value 1e-10 were further analysed by BLASTx algorithm against *Arabidopsis *protein database, and sequences showing highest sequence homology with the corresponding *Arabidopsis *genes were selected. The sequences lacking from *Fragaria *were similarly searched from GDR Rosaceae EST database and from Rosaceae protein database at NCBI.

### Photoperiod and temperature treatments

For the analysis of environmental regulation of flowering in EB genotypes, seeds of 'Baron Solemacher', and 'Hawaii-4' were germinated in 18 h LD at 18°C. During germination, plants were illuminated using 400 W SON-T lamps (Airam) for 12 h daily (90 ± 10 μmol m^-2 ^s^-1 ^at plant height plus natural light) and incandescent lamps were used for low-intensity daylength extension (5 ± 1 μmol m^-2 ^s^-1 ^at plant height). After opening of the cotyledons plants were moved to four treatments, SD and LD (12/18 h) at low or high temperature (11/18°C), for five weeks. In LD, incandescent lamps were used for low-intensity daylength extension (5 ± 1 μmol m^-2 ^s^-1 ^at plant height) after 12 h main light period. Also photoperiods of 8 and 8 + 8 h (SD/LD) were tested, but because of very slow growth in these light treatments, longer photoperiods were selected (data not shown). SD treatments were carried out at the greenhouse using darkening curtains, while LD treatments (photoperiod 18 h) were conducted in a similar greenhouse compartment without curtains. The experiments were carried out during winter 2007 - 2008, when the natural day length was under 12 h. After treatments, plants were potted to 8 x 8 cm pots, moved to LD (18 h), and flowering time was determined as described above.

### Gene expression analysis

Total RNA from leaf and shoot apex samples was extracted with a pine tree method [74], and cDNAs were synthesized from total RNA using Superscript III RT kit (Invitrogen, Carlsbad, US) and dT_18_VN anchor primers. LightCycler 480 SYBR Green I Master kit (Roche Diagnostics, Indianapolis, US) was used to perform 15 μl real-time RT-PCR reactions in 384-well plates according to manufacturer's instructions by using Light Cycler 480 real-time PCR system (Roche Diagnostics). PCR primers with T_m _value of 60°C were used (Table 5). Three biological replicates were analysed for shoot apex samples from different developmental stages (Figure 4), and two biological replicates were used for pooled shoot apex and leaf samples (Table 6).

## Authors' contributions

TH, KM and PE designed all experiments. PE coordinated the study and helped to draft the manuscript. TH run the real-time PCR analysis, performed flowering gene searches from sequence databases, and drafted the manuscript together with KM. KM constructed the subtracted cDNA libraries and performed bioinformatics analysis together with KF. KF also helped to draft the manuscript. MR participated in flowering time analysis and sampling of shoot apices. PA and LP were responsible for the EST sequencing. All authors read and approved the final manuscript.

## Supplementary Material

Additional file 1 **Functional classification of ESTs from EB and SD genotypes**. The percentage of gene hits in different FunCat classes in two cDNA libraries prepared in this study is shown. Same gene may be classified in one or several classes. WT and EB libraries were prepared from SD and everbearing genotypes, respectively. Click here for file

Additional file 2 **Complete list of flowering time genes searched**. Genes belonging to different flowering pathways are listed in separate sheets of .xls file. Homologous sequences found in *Fragaria *are indicated. Moreover, corresponding Rosaceae sequences were searched, if *Fragaria *sequence was not found. GenBank EST sequence number or Genome Database for Rosaceae contig number is given for *Fragaria *and Rosaceae ESTs and contigs, respectively. Click here for file

Additional file 3 **The stability of control genes used in this study**. Ct values of *FVE*, *MSI *and *UBI *in the leaf samples collected at four leaf stage (**a**) and in the pooled shoot apex samples (**b**). Same plant material of SD and EB ('Baron Solemacher') genotypes was used than in Table 6. Panel **c**: Ct values of *UBI *in the shoot apex samples of SD and EB genotypes at different developmental stages. Values are means (± standard deviation) of two (a, b) or three (c) biological and three technical replicates. One μg of total RNA was used for cDNA synthesis for each sample. Different Ct values of *UBI *in shoot apex samples in figures b and c are due to different cDNA dilutions used for PCR. Click here for file

## References

[B1] Ausín I, Alonso-Blanco C, Martinez-Zapater M (2005). Environmental regulation of flowering. Int J Dev Biol.

[B2] Putterill J, Laurie R, Macknight R (2004). It's time to flower: the genetic control of flowering time. Bioessays.

[B3] Simpson GG (2004). The autonomous pathway: epigenetic and post-transcriptional gene regulation in the control of Arabidopsis flowering time. Curr Opinion Plant Biol.

[B4] Imaizumi T, Kay SA (2006). Photoperiodic control of flowering: not only by coincidence. Trends Plant Sci.

[B5] Strayer C, Oyama T, Schultz TF, Raman R, Somers DE, Más P, Panda S, Kreps JA, Kay SA (2000). Cloning of the *Arabidopsis *clock gene *TOC1*, an autoregulatory response regulator homolog. Science.

[B6] Wang ZY, Tobin EM (1998). Constitutive expression of the *CIRCADIAN CLOCK ASSOCIATED 1 *(*CCA1*) gene disrupts circadian rhytms and suppresses its own expression. Cell.

[B7] Schaffer R, Ramsay N, Samach A, Corden S, Putterill J, Carré IA, Coupland G (1999). The *late elongated hypocotyl *mutation of *Arabidopsis *disrupts circadian rhythms and the photoperiodic control of flowering. Cell.

[B8] Suárez-López P, Wheatley K, Robson F, Onouchi H, Valverde F, Coupland G (2001). *CONSTANS *mediates between the circadian clock and the control of flowering in *Arabidopsis*. Nature.

[B9] Yanovsky MJ, Kay SA (2002). Molecular basis of seasonal time measurement in Arabidopsis. Nature.

[B10] Abe M, Kobayashi Y, Yamamoto S, Daimon Y, Yamaguchi A, Ikeda Y, Ichinoki H, Notaguchi M, Goto K, Araki T (2005). FD, a bZIP protein mediating signals from the floral pathway integrator FT at the shoot apex. Science.

[B11] Corbesier L, Vincent C, Jang S, Fornara F, Fan Q, Searle I, Giakountis A, Farrona S, Gissot L, Turnbull C, Coupland G (2007). FT protein movement contributes to long-distance signalling in floral induction of Arabidopsis. Science.

[B12] Parcy M (2005). Flowering: a time for integration. Int J Dev Biol.

[B13] Searle I, He Y, Turck F, Vincent C, Fornara F, Krober S, Amasino RA, Coupland G (2006). The transcription factor FLC confers a flowering response to vernalization be repressing meristem competence and systemic signalling in *Arabidopsis*. Genes Dev.

[B14] Johanson U, West J, Lister C, Michaels S, Amasino R, Dean C (2000). Molecular analysis of *FRIGIDA*, a major determinant of natural variation in Arabidopsis flowering time. Science.

[B15] Michaels SD, Bezerra IC, Amasino RM (2004). *FRIGIDA*-related genes are required for the winter-annual habit in *Arabidopsis*. PNAS.

[B16] Noh Y, Amasino RS (2003). *PIE1*, an ISWI family gene, is required for *FLC *activation and floral repression in *Arabidopsis*. Plant Cell.

[B17] He Y, Doyle MR, Amasino RM (2004). PAF1-complex-mediated histone methylation of *FLOWERING LOCUS C *chromatin is required for the vernalization-responsive, winter-annual habit in *Arabidopsis*. Genes Dev.

[B18] Zhang H, Ransom C, Ludwig P, van Nocker S (2003). Genetic analysis of early flowering mutants in *Arabidopsis *defines a class of pleiotropic developmental regulator required for expression of the flowering-time switch *Flowering Locus C*. Genetics.

[B19] Wood CC, Robertson M, Tanner G, Peacock WJ, Dennis ES, Helliwell CA (2006). The *Arabidopsis thaliana *vernalization response requires a Polycomb-like protein complex that also includes VERNALIZATION INSENSITIVE 3. PNAS.

[B20] Sung S, Amasino RM (2004). Vernalization in Arabidopsis thaliana is mediated by the PHD finger protein VIN3. Nature.

[B21] Quesada V, Dean C, Simpson GG (2005). Regulated RNA processing in the control of *Arabidopsis *flowering. Int J Dev Biol.

[B22] Jiang D, Yang W, He Y, Amasino RM (2007). *Arabidopsis *relatives of the human lysine-specific demethylase 1 repress the expression of *FWA *and *FLOWERING LOCUS C *and thus promote the floral transition. Plant Cell.

[B23] He Y, Michaels SD, Amasino RM (2003). Regulation of flowering time by histone acetylation in Arabidopsis. Science.

[B24] Ausín I, Alonso-Blanco C, Jarillo JA, Ruiz-García L, Martínez-Zapater JM (2004). Regulation of flowering time by FVE, a retinoblastoma-associated protein. Nat Genet.

[B25] Gogal GFW, Sheldon CC, Gubler F, Moritz T, Bagnall DJ, MacMillan CP, Li SF, Parish RW, Dennis ES, Weigel D, King RW (2001). GAMYB-like genes, flowering, and gibberellin signaling in *Arabidopsis*. Plant Physiol.

[B26] Jonkers H (1965). On the flower formation, the dormancy and the early forcing of strawberries. Thesis.

[B27] Heide O, Sønsteby A (2007). Interactions of temperature and photoperiod in the control of flowering of latitudinal and altitudinal populations of wild strawberry (*Fragaria vesca*). Physiol Plant.

[B28] Heide O (1977). Photoperiod and temperature interactions in growth and flowering of strawberry. Physiol Plant.

[B29] Guttridge CG, Halevy A (1985). Fragaria × ananassa. CRC Handbook of Flowering.

[B30] Konsin M, Voipio I, Palonen P (2001). Influence of photoperiod and duration of short-day treatment on vegetative growth and flowering of strawberry (*Fragaria *× *ananassa *Duch.). J Hort Sci Biotech.

[B31] Hytönen T, Palonen P, Mouhu K, Junttila O (2004). Crown branching and cropping potential in strawberry (*Fragaria *× *ananassa*, Duch.) can be enhanced by daylength treatments. J Hort Sci Biotech.

[B32] Darrow GM (1966). The strawberry History, breeding and physiology.

[B33] Durner EF, Barden JA, Himelrick DG, Poling EB (1984). Photoperiod and temperature effects on flower and runner development in day-neutral, junebearing and everbearing strawberries. J Amer Soc Hort Sci.

[B34] Sønsteby A, Heide OM (2007). Long-day control of flowering in everbearing strawberries. J Hort Sci Biotech.

[B35] Sønsteby A, Heide OM (2008). Long-day rather than autonomous control of flowering in the diploid everbearing strawberry *Fragaria vesca ssp. semperflorens*. J Hort Sci Biotech.

[B36] Albani M, Battey NH, Wilkinson MJ (2004). The development of ISSR-derived SCAR markers around the *SEASONAL FLOWERING LOCUS *(*SFL*) in *Fragaria*. Theor Appl Gen.

[B37] Guttridge CG (1959). Further evidence for a growth-promoting and flower-inhibiting hormone in strawberry. Annals Bot.

[B38] Thompson PA, Guttridge CG (1959). Effect of gibberellic acid on the initiation of flowers and runners in the strawberry. Nature.

[B39] Guttridge CG, Thompson PA (1963). The effects of gibberellins on growth and flowering of *Fragaria *and *Duchesnea*. J Exp Bot.

[B40] Brown T, Wareign PF (1965). The genetic control of the everbearing habit and three other characters in varieties of *Fragaria vesca*. Euphytica.

[B41] Battey N, Miere P, Tehranifar A, Cekic C, Taylor S, Shrives K, Hadley P, Greenland A, Darby J, Wilkinson M, Cockshull KE, Gray D, Seymour GB, Thomas B (1998). Genetic and environmental control of flowering in strawberry. Genetic and Environmental Manipulation of Horticultural Crops.

[B42] Diatchenko L, Lau YF, Campbell AP, Chenchik A, Moqadam F, Huang B, Lukyanov S, Lukyanov K, Gurskaya N, Sverdlov ED, Siebert PD (1996). Suppression subtractive hybridization: a method for generating differentially regulated or tissue-specific cDNA probes and libraries. PNAS.

[B43] Thomas B (2006). Light signals and flowering. J Exp Bot.

[B44] Stewart P (2007). Molecular characterization of photoperiodic flowering in strawberry (*Fragaria *sp.). PhD thesis.

[B45] Fowler S, Lee K, Onouchi H, Samach A, Richardson K, Morris B, Coupland G, Putterill J (1999). GIGANTEA: a circadian clock-controlled gene that regulates photoperiodic flowering in *Arabidopsis *and encodes a protein with several possible membrane-spanning domains. EMBO J.

[B46] Jang S, Marchal V, Panigrahi KCS, Wenkel S, Soppe W, Deng X, Valverde F, Coupland G (2008). *Arabidopsis *COP1 shapes the temporal pattern of CO accumulation conferring a photoperiodic flowering response. EMBO J.

[B47] Laubinger S, Marchal V, Le Gourrierec J, Wenkel S, Adrian J, Jang S, Kulajta C, Braun H, Coupland G, Hoecker U (2006). *Arabidopsis *SPA proteins regulate photoperiodic flowering and interact with floral inducer *CONSTANS *to regulate its stability. Development.

[B48] Cerdán PD, Chory J (2003). Regulation of flowering time by light quality. Nature.

[B49] Kang X, Zhou Y, Sun X, Ni M (2007). HYPERSENSITIVE TO RED AND BLUE 1 and its C-terminal regulatory function control *FLOWERING LOCUS T *expression. Plant J.

[B50] Scortecci KC, Michaels SD, Amasino RM (2001). Identification of a MADS-box gene, *FLOWERING LOCUS M*, that repress flowering. Plant J.

[B51] Reeves PA, He Y, Schmitz RJ, Amasino RM, Panella LW, Richards CM (2007). Evolutionary conservation of the *FLOWERING LOCUS C*-mediated vernalization response: evidence from the sugar beet (*Beta vulgaris*). Genetics.

[B52] Choi K, Park C, Lee J, Oh M, Noh B, Lee I (2007). *Arabidopsis *homologs of components of the SWR1 complex regulate flowering and plant development. Development.

[B53] Kim KS, Choi K, Park C, Hwanga H, Lee I (2006). *SUPPRESSOR OF FRIGIDA4*, encoding a C2H2-type zinc finger protein, represses flowering by transcriptional activation of *Arabidopsis FLOWERING LOCUS C*. Plant Cell.

[B54] Folta KM, Staton M, Stewart PJ, Jung S, Bies DH, Jesudurai C, Main D (2005). Expressed sequence tags (ESTs) and simple sequence repeat (SSR) markers from octoploid strawberry (*Fragaria *× *ananassa*). BMC Plant Biol.

[B55] Levy YY, Mesnage S, Mylne JS, Gendall AR, Dean C Multiple roles of *Arabidopsis VRN1 *in vernalization and flowering time control. Science.

[B56] Mylne JS, Barrett L, Tessadori F, Mesnage S, Johnson L, Bernatavichute VN, Jacobsen SE, Fransz P, Dean C (2006). LHP1, the *Arabidopsis *homologue of HETEROCHROMATIN PROTEIN1, is required for epigenetic silencing of *FLC*. PNAS.

[B57] Lee JH, Yoo SJ, Park SH, Hwang I, Lee JS, Ahn JH (2007). Role of SVP in the control of flowering time by ambient temperature in *Arabidopsis*. Genes Dev.

[B58] Wagner D, Sablowski RWM, Meyerowitz EM (1999). Transcriptional activation of APETALA1 by LEAFY. Science.

[B59] Turck F, Fornara F, Coupland G (2008). Regulation and identity of florigen: FLOWERING LOCUS T moves central stage. Annu Rev Plant Biol.

[B60] Zagotta MT, Hicks KA, Jacobs CI, Young JC, Hangarter RP, Meeks-Wagner D (1996). The *Arabidopsis ELF3 *gene regulates vegetative photomorphogenesis and the photoperiodic induction of flowering. Plant J.

[B61] Weebadde CK, Wang D, Finn CE, Lewers KS, Luby JJ, Bushakra J, Sjulin TM, Hancock JF (2008). Using a linkage mapping approach to identify QTL for day-neutrality in the octoploid strawberry. Plant Breed.

[B62] Ahmadi H, Bringhurst RS, Voth V (1990). Modes of inheritance of photoperiodism in *Fragaria*. J Amer Soc Hort Sci.

[B63] Shulaev V, Korban SS, Sosinski B, Abbott AG, Aldwinckle HS, Folta KM, Iezzoni A, Main D, Arús P, Dandekar AM, Lewers K, Brown SK, Davis TM, Gardiner SE, Potter D, Veilleux RE (2008). Multiple models for Rosaceae genomics. Plant Physiol.

[B64] Takada S, Goto K (2003). Terminal flower 2, an Arabidopsis homolog of heterochromatin protein 1, counteracts the activation of *Flowering locus T *by Constans in the vascular tissues of leaves to regulate flowering time. Plant Cell.

[B65] Sawa M, Nusinow DA, Kay SA, Imaizumi T (2007). FKF1 and GIGANTEA complex formation is required for day-length measurement in *Arabidopsis*. Science.

[B66] Hecht V, Foucher F, Ferrandiz C, Macknight R, Navarro C, Morin J, Vardy ME, Ellis N, Beltran J, Rameau C, Weller JL (2005). Conservation of *Arabidopsis *flowering genes in model legumes. Plant Physiol.

[B67] Alabadi D, Oyama T, Yanovsky MJ, Harmon FG, Mas P, Kay SA (2001). Reciprocal regulation between *TOC1 *and *LHY/CCA1 *within the *Arabidopsis *circadian clock. Science.

[B68] Böhlenius H (2008). Control of flowering time and growth cessation in *Arabidopsis *and *Populus *trees. PhD thesis.

[B69] Wang R, Farrona S, Vincent C, Joecker A, Schoof H, Turck F, Alonso-Blanco C, Coupland G, Albani MC (2009). *PEP1 *regulates perennial flowering in *Arabis alpina*. Nature.

[B70] Zhang X, Clarenz O, Cokus S, Bernatavichute YV, Pellegrini M, Goodrich J, Jacobsen SE (2007). Whole genome analysis of histone H3 lysine 27 trimethylation in *Arabidopsis*. PloS Biol.

[B71] Yano M, Katayose Y, Ashikari M, Yamanouchi U, Monna L, Fuse T, Baba T, Yamamoto K, Umehara Y, Nagamura Y, Sasaki T (2000). *Hd1*, a major photoperiod sensitivity quantitative trait locus in rice, is closely related to the *Arabidopsis *flowering time gene *CONSTANS*. Plant Cell.

[B72] Sargent DJ, Clarke J, Simpson DW, Tobutt KR, Arús P, Monfort A, Vilanova S, Denoyes-Rothan B, Rousseau M, Folta KM, Bassil NV, Battey NH (2006). An enhanced microsatellite map of diploid *Fragaria*. Theor Appl Genet.

[B73] Roberts AV, Blake PS, Lewis R, Taylor JM, Dunstan DI (1999). The effect of gibberellins on flowering in roses. J Plant Growth Regul.

[B74] Monte D, Somerville S, Bowtell D, Sambrook J (2002). Pine tree method for isolation of plant RNA. DNA microarrays: a molecular cloning manual.

[B75] Laitinen RAE, Immanen J, Auvinen P, Rudd S, Alatalo E, Paulin L, Ainasoja M, Kotilainen M, Koskela S, Teeri TH, Elomaa P (2005). Analysis of the floral transcriptome uncovers new regulators of organ determination and gene families related to flower organ differentiation in *Gerbera hybrida *(Asteraceae). Genome Res.

[B76] Tuskan GA, DiFazio S, Jansson S, Bohlmann J, Grigoriev I (2006). The genome of black cottonwood, *Populus trichocarpa *(Torr. & Gray). Science.

[B77] Childs KL, Hamilton JP, Zhu W, Ly E, Cheung F, Wu H, Rabinowicz PD, Town CD, Buell CR, Chan AP (2007). The TIGR plant transcript assemblies database. Nucleic Acids Res.

[B78] Mockler T, Yang H, Yu X, Parikh D, Cheng Y, Dolan S, Lin C (2003). Regulation of photoperiodic flowering by Arabidopsis photoreceptors. PNAS.

[B79] Guo HW, Yang WY, Mockler TC, Lin CT (1998). Regulation of flowering time by *Arabidopsis *photoreceptors. Science.

[B80] Kim W, Fujiwara S, Suh S, Kim J, Kim Y, Han L, David K, Putterill J, Nam HG, Somers DE (2007). ZEITLUPE is a circadian photoreceptor stabilized by GIGANTEA in blue light. Nature.

[B81] Kim D, Kang J, Yang S, Chung K, Song P, Park C (2002). A phytochrome-associated protein phosphatase 2A modulates light signals in flowering time control in Arabidopsis. Plant Cell.

[B82] Staiger D, Allenbach L, Salathia N, Fiechter V, Davis SJ, Millar AC, Chory J, Fankhauser C (2003). The *Arabidopsis SRR1 *gene mediates phyB signaling and is required for normal circadian clock function. Genes Dev.

[B83] Hazen SP, Schultz TF, Pruneda-Paz JL, Borevitz JO, Ecker JR, Kay SA (2005). LUX ARRHYTHMO encodes a myb domain protein essential for circadian rhythms. PNAS.

[B84] Doyle MR, Davis SJ, Bestow RM, McWatters HG, Kozma-Bognar L, Nagy F, Millar AJ, Amasino MR (2002). The *ELF4 *gene controls circadian rhythms and flowering time in *Arabidopsis thaliana*. Nature.

[B85] Nakamichi N, Kita M, Niinuma K, Ito S, Yamashino T, Mizoguchi T, Mizuno T (2007). *Arabidopsis *clock-associated pseudo-response regulators PRR9, PRR7 and PRR5 coordinately and positively regulate flowering time through the canonical CONSTANS-dependent photoperiodic pathway. Plant Cell Physiol.

[B86] Noh B, Lee S, Kim H, Yi G, Shin E, Lee M, Jung KMR, Doyle KMR, Amasino RM, Noh Y Divergent roles of a pair of homologous jumonji/zinc-finger-class transcription factor proteins in the regulation of *Arabidopsis *flowering time. Plant Cell.

[B87] Hanzawa Y, Money T, Bradley D (2005). A single amino acid converts a repressor to an activator of flowering. PNAS.

[B88] Chen M, Ni M (2006). RFI2, a RING-domain zinc finger protein, negatively regulates *CONSTANS *expression and photoperiodic flowering. Plant J.

[B89] Cai X, Ballif J, Endo S, Davis E, Liang M, Chen D, DeWald D, Kreps J, Zhu T, Wu Y (2007). A putative CCAAT-binding transcription factor is a regulator of flowering timing in *Arabidopsis*. Plant Physiol.

[B90] Pien S, Fleury DF, Mylne JS, Crevillen P, Inzé D, Avramova Z, Dean C, Grossniklaus U (2008). ARABIDOPSIS THITHORAX1 dynamically regulates *FLOWERING LOCUS C *activation via histone 3 lysine 4 trimethylation. Plant Cell.

[B91] Zhang H, van Nocker S (2002). The *VERNALIZATION INDEPENDENCE 4 *gene encodes a novel regulator of *FLOWERING LOCUS C*. Plant J.

[B92] Gendall AR, Levy YY, Wilson A, Dean C (2001). The *VERNALIZATION 2 *gene mediates the epigenetic regulation of vernalization in *Arabidopsis*. Cell.

[B93] Chanvivattana Y, Bishopp A, Schubert D, Stock C, Moon Y, Sung ZR, Goodrich J (2004). Interaction of Polycomb-group proteins controlling flowering in *Arabidopsis*. Development.

[B94] MacKnight R, Bancroft I, Page T, Lister C, Schmidt R, Love K, Westphal L, Murphy G, Sherson S, Cobbett C, Dean C (1997). *FCA*, a gene controlling flowering time in *Arabidopsis thaliana *encodes a protein containing RNA binding domains. Cell.

[B95] Schomburg FM, Patton DA, Meinke DW, Amasino RM (2001). *FPA*, a gene involved in floral induction in *Arabidopsis thaliana*, encodes a protein containing RNA-recognition motifs. Plant Cell.

[B96] Lim MH, Kim J, Kim YS, Chung KS, Seo YH, Lee I, Kim J, Hong CB, Kim HJ, Park CM (2004). A new *Arabidopsis thaliana *gene, *FLK*, encodes a RNA binding protein with K homology motifs and regulates flowering time via *FLOWERING LOCUS C*. Plant Cell.

[B97] Simpson GG, Dijkwel PP, Quesada V, Henderson I, Dean C (2003). FY is a RNA 3'end-processing factor that interacts with FCA to control the *Arabidopsis thaliana *floral transition. Cell.

[B98] Wang X, Zhang Y, Ma Q, Zhang Z, Xue Y, Bao S, Chong K (2007). SKB1-mediated symmetric dimethylation of histone H4R3 controls flowering time in *Arabidopsis*. EMBO J.

[B99] Lee I, Aukerman MJ, Gore SL, Lohman KN, Michaels SD, Weaver LM, John MC, Feldmann KA, Amasino RM (1994). Isolation of *LUMINIDEPENDENS*: a gene involved in the control of flowering time in *Arabidopsis thaliana*. Plant Cell.

[B100] Cheng H, Qin L, Lee S, Fu X, Richards DE, Cao D, Luo D, Harberd NP, Peng J (2004). Gibberellin regulates *Arabidopsis *floral development via suppression of DELLA protein function. Development.

[B101] Tseng TS, Salomé PA, McClung CR, Olszewski NE (2004). SPINDLY and GIGANTEA interact and act in *Arabidopsis thaliana *pathways involved in light responses, flowering and rhythms in leaf movements. Plant Cell.

[B102] Magome H, Yamaguchi S, Hanada A, Kamiya Y, Oda K (2004). *Dwarf and delayed-flowering 1*, a novel *Arabidopsis *mutant deficient in gibberellin biosynthesis because of overexpression of putative AP2 transcription factor. Plant J.

[B103] Kania T, Russenberger D, Peng S, Apel K, Melzer S (1997). FPF1 promotes flowering in *Arabidopsis*. Plant Cell.

[B104] Aukerman MJ, Sakai H (2003). Regulation of flowering time and floral organ identity by a microRNA and its *APETALA2-like *target genes. Plant Cell.

