# Supplementary material for: Identification of flowering genes in strawberry, a perennial SD plant
Source: BMC Plant Biol. 2009 Sep 28;9:122. doi: 10.1186/1471-2229-9-122 (PMC2761920; doi:10.1186/1471-2229-9-122)
Supplement: Additional file 1 — Functional classification of ESTs from EB and SD genotypes. The percentage of gene hits in different FunCat classes in two cDNA libraries prepared in this study is shown. Same gene may be classified in one or several classes. WT and EB libraries were prepared from SD and everbearing genotypes, respectively. [file 1471-2229-9-122-S1.PDF]

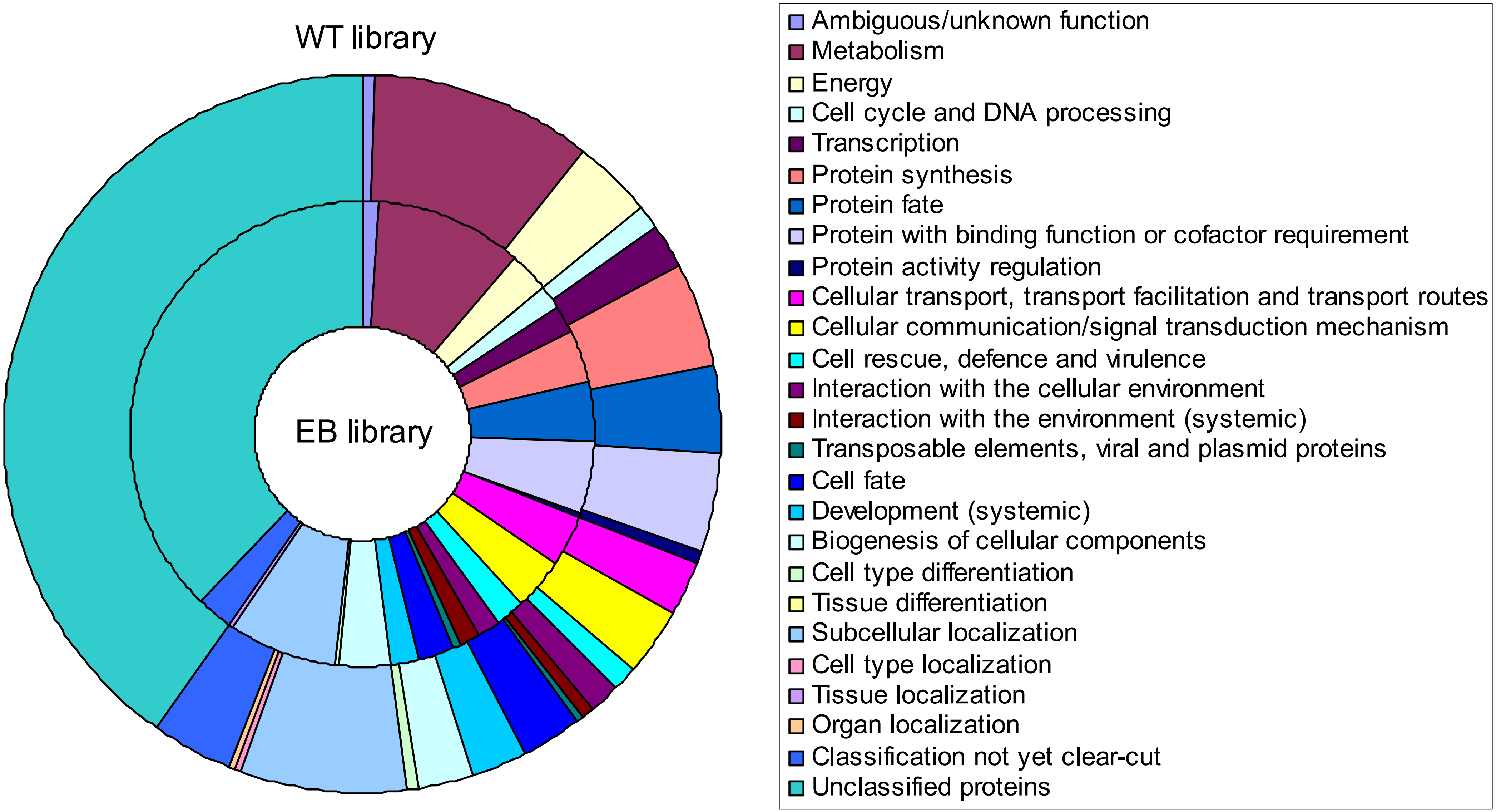
WT library
EB library
Ambiguous/unknown function
Metabolism
Energy
Cell cycle and DNA processing
Transcription
Protein synthesis
Protein fate
Protein with binding function or cofactor requirement
Protein activity regulation
Cellular transport, transport facilitation and transport routes
Cellular communication/signal transduction mechanism
Cell rescue, defence and virulence
Interaction with the cellular environment
Interaction with the environment (systemic)
Transposable elements, viral and plasmid proteins
Cell fate
Development (systemic)
Biogenesis of cellular components
Cell type differentiation
Tissue differentiation
Subcellular localization
Cell type localization
Tissue localization
Organ localization
Classification not yet clear-cut
Unclassified proteins
