# Supplementary figures and images for: Identification of flowering genes in strawberry, a perennial SD plant
Source: BMC Plant Biol. 2009 Sep 28;9:122. doi: 10.1186/1471-2229-9-122 (PMC2761920; doi:10.1186/1471-2229-9-122)

## Slide 1
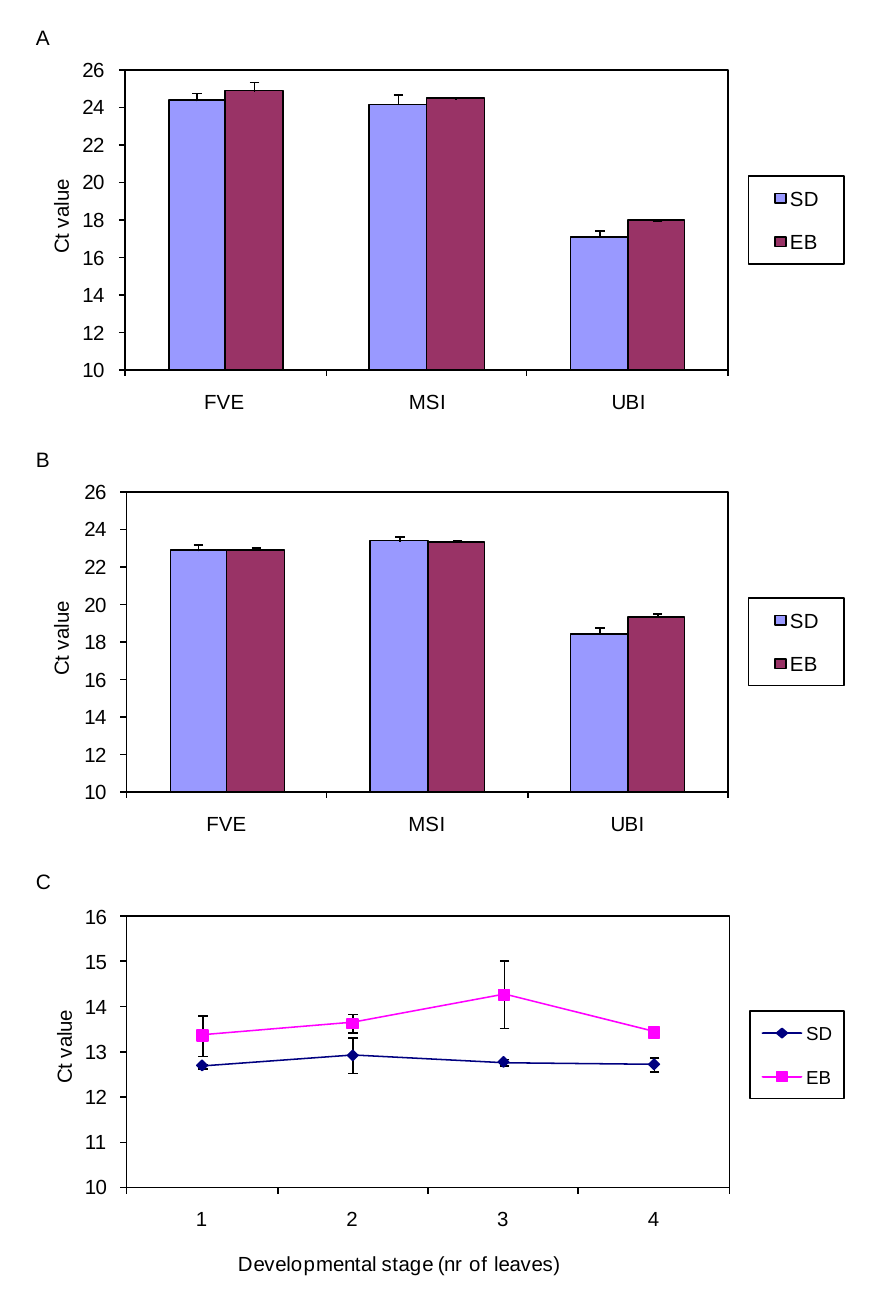

A
B
C

Supplement: Additional file 3 — The stability of control genes used in this study. Ct values of FVE, MSI and UBI in the leaf samples collected at four leaf stage (a) and in the pooled shoot apex samples (b). Same plant material of SD and EB ('Baron Solemacher') genotypes was used than in Table 6. Panel c: Ct values of UBI in the shoot apex samples of SD and EB genotypes at different developmental stages. Values are means (± standard deviation) of two (a, b) or three (c) biological and three technical replicates. One μg of total RNA was used for cDNA synthesis for each sample. Different Ct values of UBI in shoot apex samples in figures b and c are due to different cDNA dilutions used for PCR. [file 1471-2229-9-122-S3.PPT]
